# Nano spin-diodes using FePt-NDs with huge on/off current ratio at room temperature

**DOI:** 10.1038/srep33409

**Published:** 2016-09-12

**Authors:** Katsunori Makihara, Takeshi Kato, Yuuki Kabeya, Yusuke Mitsuyuki, Akio Ohta, Daiki Oshima, Satoshi Iwata, Yudi Darma, Mitsuhisa Ikeda, Seiichi Miyazaki

**Affiliations:** 1 Graduate School of Engineering, Nagoya University, Aichi, Japan; 2 Department of Physics, Institut Teknologi Bandung, Bandung, Indonesia; 3 Singapore Synchrotron Light Source, National University of Singapore, Singapore

## Abstract

Spin transistors have attracted tremendous interest as new functional devices. However, few studies have investigated enhancements of the ON/OFF current ratio as a function of the electron spin behavior. Here, we found a significantly high spin-dependent current ratio—more than 10^2^ at 1.5 V—when changing the relative direction of the magnetizations between FePt nanodots (NDs) and the CoPtCr-coated atomic force microscope (AFM) probe at room temperature. This means that ON and OFF states were achieved by switching the magnetization of the FePt NDs, which can be regarded as spin-diodes. The FePt magnetic NDs were fabricated by exposing a bi-layer metal stack to a remote H_2_ plasma (H_2_-RP) on ~1.7 nm SiO_2_/Si(100) substrates. The ultrathin bi-layers with a uniform surface coverage are changed drastically to NDs with an areal density as high as ~5 × 10^11^ cm^−2^. The FePt NDs exhibit a large perpendicular anisotropy with an out-of-plane coercivity of ~4.8 kOe, reflecting the magneto-crystalline anisotropy of (001) oriented L1_0_ phase FePt. We also designed and fabricated double-stacked FePt-NDs with low and high coercivities sandwiched between an ultra-thin Si-oxide interlayer, and confirmed a high ON/OFF current ratio when switching the relative magnetization directions of the low and high coercivity FePt NDs.

The spin transistor, whose ON current is controlled both by the gate/base signal and the relative magnetization direction of two ferromagnets, is considered to be one of the future devices for beyond-CMOS computing^1^,^2^,^3^,^4^,^5^,^6^,^7^,^8^,^9^,^10^,^11^. The spin transistor utilizes the magnetization of one of the ferromagnets as nonvolatile memory data, and thus it has the potential to work as a reconfigurable logic, which is beneficial in reducing the power consumption and device count on chips. However, from the application point of view, the spin transistor requires a high magneto-current ratio when switching the magnetization of the ferromagnet as well as a large ON/OFF ratio when the gate/base signal is applied. Most importantly, a ferromagnet that will store data is required to have a strong magnetic anisotropy for long-time data retention^12^,^13^,^14^,^15^,^16^,^17^,^18^. In this paper, we report on spin diodes comprising FePt nanodots (NDs) with a giant magneto current ratio. The FePt is known to have a huge magneto-crystalline anisotropy, and exhibits sufficient thermal stability even in ~3 nm sized NDs^19^,^20^,^21^,^22^,^23^.

Metallic NDs have received much attention because of their potential application to charge storage devices^24^,^25^,^26^,^27^,^28^,^29^,^30^,^31^,^32^,^33^,^34^. The charging and discharging characteristics of metallic NDs through an ultrathin oxide layer depend on their electrostatic potential. So far, we reported that Ni-, Co-, Pt-, and Fe-NDs with an areal density as high as ~10^11^ cm^−2^ were formed on ultrathin SiO_2_ by exposing an ultrathin metal layer to a remote H_2_ plasma (H_2_-RP) without external heating, and demonstrated their charge storage properties^35^,^36^,^37^,^38^,^39^,^40^. More recently, we reported on the formation of magnetic NDs made of CoPt alloy formed by exposing a bi-layer metal stack to a remote H_2_ plasma (H_2_-RP) and characterized their magnetization properties^41^. In this work, we fabricated high density L1_0_-ordered FePt-NDs on ultrathin SiO_2_/Si(100) and measured the local current-voltage (*I*-*V*) characteristics by using a CoPtCr-coated atomic force microscope (AFM) probe under the application of an external magnetic field. We found that the threshold voltage for a current to flow was controlled by the relative direction of the magnetizations of the FePt and CoPtCr, which indicates that the FePt NDs is a promising candidate for the gate material of a spin diode/transistor that exhibits quite a large magneto-current ratio as well as a large ON/OFF ratio.

## Results

### Formation of FePt-nanodots

**Figure 1:**
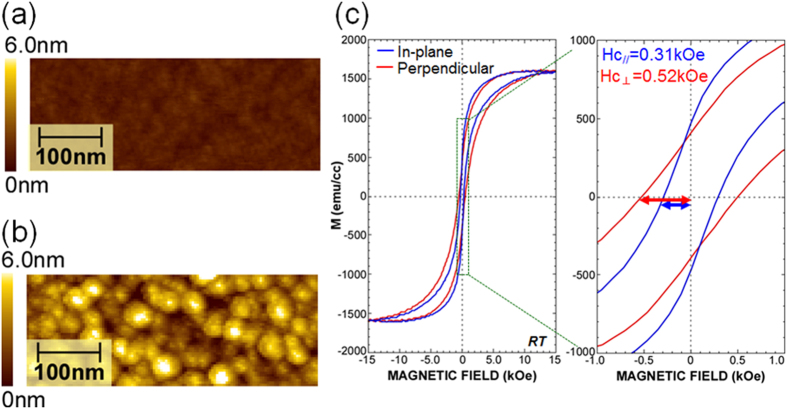
L1_0_-ordering FePt-NDs. (**a,b**) AFM topographic images before and after exposing the Pt/Fe bi-layer stack structures on SiO_2_ to H_2_-RP. (**c**) *M*-*H* curves of FePt-NDs measured applying a magnetic field along the in-plane and out-of-plane directions at room temperature. In-plane and out-of-plane coercivities were 0.31 and 0.52 kOe, respectively.

Figure 1(a,b) show atomic force microscope (AFM) images for an as-evaporated Pt (1.7 nm)/Fe (1.4 nm) bilayer on SiO_2_ and after H_2_-RP exposure, respectively, where AFM topographic images were measured with a scanning area as narrow as 500 × 500 nm^2^ to determine each dot size. The images for the as-evaporated bi-layer stack structure showed a fairly smooth surface morphology with a small root-mean-square (RMS) roughness of ~0.24 nm, being almost identical to that for the as-grown SiO_2_ surface. The result confirms uniform surface coverage with the ultrathin Pt/Fe bi-layer. By exposing the Pt/Fe bi-layer on the SiO_2_ to H_2_-RP for 10 min, the RMS roughness was increased by a factor of ~6.5, and NDs with an areal density as high as ~4.8 × 10^11^ cm^−2^ were formed. An average height of ~8.7 nm was observed (see Fig. 1(b)), which was determined by the dot height distribution (details are presented in Supplementary Fig. S1). The in-plane and out-of-plane hysteresis loops for the FePt-NDs were characterized by an alternating gradient field magnetometer at room temperature as shown in Fig. 1(c). Note that the hysteresis loops for the Pt/Fe bi-layer (not shown in this paper) showed an easy plane of magnetization along the film plane and exhibited almost zero coercivity in both the perpendicular and in-plane loops. In contrast, the in-plane and out-of-plane loops for the FePt-NDs became closer to each other, and coercivities of 0.31 and 0.52 kOe were confirmed for the in-plane and out-of-plane loops, respectively. This indicates that a perpendicular magnetic anisotropy is induced in the FePt-NDs after exposure to H_2_-RP. The crystalline structure of the FePt-NDs was examined from the X-ray diffraction (XRD) pattern, in which strong (001) superlattice and (002) fundamental peaks were seen in the L1_0_-FePt, while no characteristic diffraction peaks except for the Si (001) substrate were observable in the XRD pattern of the Pt/Fe bilayer (see Supplementary Fig. S2). Electrical isolation between the FePt-alloy NDs was confirmed by changes in surface potential due to electron charging of the dots as discussed later.

### Local current-voltage characteristics of the FePt-nanodot

**Figure 2:**
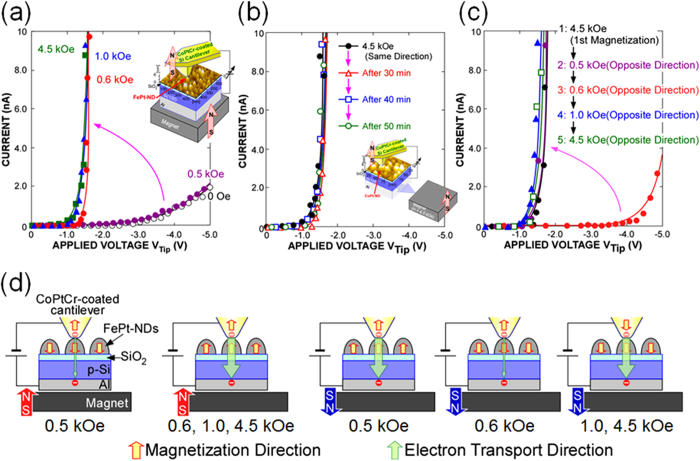
Local I-V characteristics of FePt-NDs. (**a**) *I*-*V* characteristics measured using a CoPtCr-coated AFM-tip with and without application of a magnetic field in the same direction as the initial tip magnetization. Measurement setup is shown in inset. (**b**) *I*-*V* characteristics measured after 30 to 50 min passed after removing the external field. c, *I*-*V* characteristics measured under the application of a magnetic field opposite to the first magnetization direction at 4.5 kOe. (**d**) Schematics of the variation in the directions of magnetization of the FePt-NDs and CoPtCr-cantilever with the application of an external field.

To evaluate the local electron transport properties of the FePt-alloy NDs, a CoPtCr- or Rh-coated Si cantilever was positioned in contact with the sample surface and kept at a certain position, and the local *I*-*V* characteristics were measured at room temperature. A typical *I*-*V* characteristic for a ND using a CoPtCr-coated tip is shown in Fig. 2(a), where electrons are injected from the tip. The magnetization of the tip was initially set along the upward direction, and upward (positive) and downward (negative) external magnetic fields were applied to the NDs by placing an NdFeB magnet under the sample, where the magnetic field strength was calibrated by a gauss meter (see inset in Fig. 2(a)). Before the application of the external field to the sample, the current level increased slightly for a tip bias over ~−3.0 V. When a positive field of 0.5 kOe was applied to the sample in the same direction as the initial tip magnetization, there was no significant change in the *I*-*V* curve from the case before application of the field. However, a distinct increase in the current level was detected when a positive field of 0.6 kOe was applied, that is, the threshold voltage needed for a current of 1 nA to flow decreased from −4.3 to −1.2 V. A further increase in the positive field resulted in no obvious changes in the *I*-*V* curve. When the CoPtCr-coated tip was replaced by an Rh-coated tip, almost no change was detected in the *I*-*V* curve with the application of a magnetic field of 4.5 kOe, irrespective of the field direction (see Supplementary Fig. S3). In addition, no significant changes in the *I*-*V* curve were obtained when the same experiments were performed for Pt-NDs (see Supplementary Fig. S4). From these experiments, in discussing *I*-*V* properties between the FePt NDs and the CoPtCr-coated tip, we can rule out the influence of the magnetic force acting on the tip under the external field and any magneto-static interaction between the tip and the dot. Actually, from the force curve measurements with and without application of a magnetic force, no changes in the force curve were detected (see Supplementary Fig. S5). It is interesting to note that no significant change in the *I*-*V* curve was confirmed even after the external magnetic field was removed after application of the initial field of 4.5 kOe (Fig. 2(b)). We also confirmed a remarkable increase in the threshold voltage from −1.2 V to −4.3 V when an external field of −0.6 kOe was applied in the opposite direction. Further increases in the external field to more than 1.0 kOe in the opposite direction reduced the threshold voltage again, as shown in Fig. 2(c). We confirmed a reversal of the tip magnetization with a magnetic field of 1.0 kOe by measuring the reversed contrast in the MFM image of a floppy disk with the tip exposed to a field of 1.0 kOe. Based on these results, we summarized the direction of magnetization of the dots and the tip under the application of a magnetic field, namely the impact of the application of a magnetic field on the local electron transport characteristics between the FePt-NDs and CoPtCr-coated tip, as illustrated schematically in Fig. 2(d).

### Charge storage characteristics of FePt-nanodots

**Figure 3:**
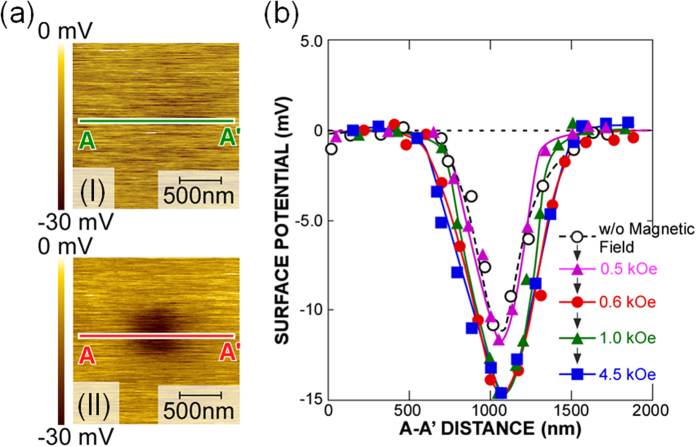
Surface potential of FePt-NDs. (**a**) Surface potential images before (I) and after (II) the application of a bias of −2.0 V in the central part of the 500 × 500 nm^2^ area. (**b**) Cross-sectional potential profiles along the line A-A′ shown in the image in Fig. (a) with application of a magnetic field.

In order to understand the significant variation in the threshold voltage with the relative direction of magnetization between the CoPtCr-tip and FePt-NDs, changes in the surface potential of the FePt-NDs after electron charging the dots with and without application of a magnetic field were measured by Kelvin-probe force microscopy (KFM), as shown in Fig. 3. For the surface potential measurements shown in Fig. 3, electrons were injected into many dots over an area of 500 × 500 nm^2^ and surface potential images were taken with a large scanning area of 2000 × 2000 nm^2^ to evaluate carrier migration between neighboring dots. Without any bias applied to the sample surfaces, a uniform surface potential image was observed (Fig. 3(a)). When the surface was scanned with an Rh-coated AFM tip biased at −2.0 V with respect to the substrate in the tapping mode, a distinct decrease in the surface potential of ~10 mV, which is associated with a negative charge on the sample surface, was observed in the corresponding area (Fig. 3(b)). However, in the unbiased area, no change in the surface potential was detectable. This result suggests that each dot was electrically isolated and the dots in the corresponding area store electrons injected from the Rh-coated tip. In addition, after charging in the 500 × 500 nm^2^ region, no lateral spreading of stored charges was observable. The fact that no electrons spread laterally outside the charged region can be explained by the fact that charged electrons are stored stably in the dots with no tunneling, either through the bottom oxide to the substrate or to neighboring dots. It was also confirmed that there were no changes in the topographic images with electron injection. On the other hand, prior to the H_2_ plasma treatment for the bi-layer stack structures, no change in the surface potential was observable when applying tip biases, because of the highly electrically conductive surface. These results indicate that electrons are injected into the electrically isolated FePt NDs formed by the H_2_-RP exposure. It is interesting to note that a distinct increase in the surface potential by a factor of ~1.5 was observed when a positive magnetic field of 0.6 kOe was applied to the sample in the same direction as the initial tip magnetization, although when applying a magnetic field of below 0.6 kOe, there was no significant change in the surface potential from the case without application of the magnetic field.

### Local current-voltage characteristics of doubly-stacked FePt-nanodots

**Figure 4:**
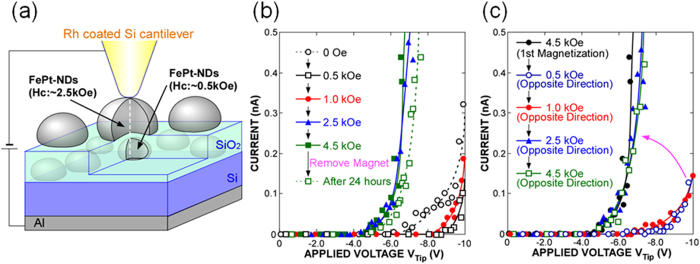
Local I-V characteristics of doubly-stacked FePt-NDs structures. (**a**) Schematic of the doubly-stacked FePt-NDs structures. (**b**) Local *I*-*V* characteristics of doubly-stacked FePt-NDs structures measured using a Rh-coated AFM tip with and without application of a magnetic field. (**c**) Local I-V characteristics of doubly-stacked FePt-NDs structures obtained with an Rh-coated AFM-tip with application of a magnetic field opposite in direction to the first field at 4.5 kOe.

We then studied doubly-stacked FePt-NDs with lower and upper dots of different sizes as shown in Fig. 4(a). To investigate the local electron transport properties of the doubly-stacked FePt-NDs structures, a non-magnetic Rh-coated Si cantilever was positioned in contact with the sample surface and kept in the same position, and the *I*-*V* characteristics were measured at room temperature. The local *I*-*V* characteristics in Fig. 4(b) show that the current level increased slightly at a tip bias of over ~−6.0 V without the application of any external magnetic field. When applying a field of 0.5 kOe, the current level decreased slightly, namely, the threshold voltage at 0.05 nA increased from −8.0 to −9.7 V. When the magnetic field was increased from 0.5 kOe to 1.0 kOe, there was no obvious change in the *I*-*V* curve. However, a distinct reduction in the threshold voltage was observed when the magnetic field was increased to 2.5 kOe, even though a non-magnetic Rh-coated tip was used. It is interesting to note that no significant change in the *I*-*V* curve was confirmed after a lapse of 24 hours without a magnetic field as shown in Fig. 4(b). When the magnetic field was increased to 0.5 kOe in the opposite direction, a marked increase in the threshold voltage was observed again, as shown in Fig. 4(c). Then, a decrease in the threshold voltage was confirmed after increasing the magnetic field to more than 2.5 kOe. When the upper and lower FePt-NDs had parallel magnetizations, both upper and lower FePt-NDs were confirmed to be electrically charged, while predominantly only the upper FePt-NDs were charged when they were antiparallel (not shown in the figure).

## Discussion

The formation of the NDs from the Pt/Fe bilayer stack shown in Fig. 1(b) suggests that the H_2_-RP exposure promotes Pt-Fe alloying with agglomeration by the cohesive action of Pt and Fe atoms on the SiO_2_ surface. In fact, the NDs exhibit a perpendicular magnetic anisotropy as shown in Fig. 1(c), while the Pt/Fe bilayer stack has an easy plane of magnetization along the film plane. The perpendicular anisotropy results from the magneto-crystalline anisotropy of the L1_0_ phase FePt alloy. The L1_0_-FePt shows a strong magneto-crystalline anisotropy with an easy axis along the *c*-axis. Formation of the (001) oriented L1_0_-FePt was confirmed by the X-ray diffraction (XRD) pattern (see Supplementary Fig. S2). Considering the fact that the temperature of the Pt and Fe foils was raised during H_2_-RP exposure up to ~400 °C^39^ and ~460 °C^40^ respectively, formation of the L1_0_-FePt-NDs can be attributed to local heating of the sample surface, which is caused by the recombination of atomic hydrogen on the Pt and FePt alloy surfaces.

In the measurement of the local *I*-*V* characteristics shown in Fig. 2, the contact between the FePt-ND and the CoPtCr-coated tip can be considered as a perpendicular magnetic tunnel junction. Therefore, the significant change in the threshold voltage of the *I*-*V* curve when applying an external field is due to the change in the electron transport property depending on the relative direction of magnetization of the FePt NDs and CoPtCr-coated tip. The change in the threshold voltage occurred at a magnetic field of 0.6 kOe, which is consistent with the out-of-plane coercivity of the FePt-NDs shown Fig. 1(c). This means that ON and OFF states were achieved by switching the magnetization of the FePt NDs which can be regarded as the gate of a spin diode. The ON and OFF states can be retained without an external magnetic field as shown in Fig. 2, because the conductivity between the FePt NDs and CoPtCr-coated tip is determined by the relative directions of the magnetization along the easy axis. However, the observed changes in the threshold voltage cannot be explained only by spin dependent tunneling due to spin-split density of states of CoPtCr-tip and FePt-NDs. To explain the significantly large on/off current ratio in Fig. 2 only by the spin dependent tunneling, the spin polarizations of the FePt-NDs and CoPtCr-tip must be over 0.99, which is not to be expected in FePt and CoPtCr.

The charged state of the FePt-NDs, which was evaluated by measuring the surface potential using a KFM, also changed corresponding to the shift in the threshold voltage in the *I*-*V* characteristics by applying an external magnetic field as shown in Fig. 3. The observed increase in the surface potential indicates that the number of electrons charged from the tip to the dot increased when the tip and FePt magnetizations were parallel compared to the case when they were antiparallel. The difference in the electron charging characteristics may originate from the different electrical conductance from the tip to the NDs just as a tunneling magneto-resistance effect, and this difference in the electron charging characteristics is considered to be the origin of the significant variation in the threshold voltage with the relative direction of magnetization between the tip and FePt-NDs as discussed and shown in Fig. 2. However, the difference in the number of electrons stored in the NDs between parallel and antiparallel magnetization alignment of the FePt-NDs and CoPtCr-tip does not explain simply the observed threshold voltage shift. When the threshold voltage shift is approximated by Q_ND_/C_ox_, the difference in the number of electrons in an ND for the threshold voltage shift of 3.1 V observed in Fig. 2 is calculated to be 52, even for the smallest ND in a size distribution with a dot height of 6.5 nm (see supplementary Fig. S1). Here, Q_ND_ is the amount of charge in an ND, and C_ox_ is the capacitance of the 1.7 nm-thick SiO_2_ layer between the ND and Si substrate, which is treated as a cylindrical parallel plate capacitor with a diameter of the hemisphere containing the ND, ignoring the edge effect. Since the thickness of the SiO_2_ between the ND and Si substrate is as thin as 1.7 nm, such a large number of electrons cannot be retained stably in an ND. Therefore, the electron storage in the NDs can only partially explain the observed threshold shift. Although the reason why the number of stored electrons in the FePt-ND is dependent on the relative magnetization direction between the FePt-NDs and CoPtCr-tip is still unclear, one of possible mechanisms to explain such a huge on/off current ratio is a synergistic effect of the tunnel magnetoresistance and the Coulomb blockade. The tunnel magnetoresistance will cause the difference of the contact resistance between tip and FePt-NDs and partly contribute to the difference of the threshold voltage between parallel and antiparallel configurations. The Coulomb blockade will cause the difference of the difference of the number of charged electrons in the ND. The energy in the Coulomb blockade regime is dependent on the magnetization directions of the electrode and ferromagnetic NDs as discussed in ref. 42. Further detailed study is needed to identify the origin of the changes in the threshold voltage.

The magnetic tunnel junction was also formed by simply stacking FePt-NDs of different sizes, namely different coercivity, with an ultrathin oxide interlayer as illustrated schematically in Fig. 4(a). The difference between the coercivities of the upper and lower FePt-NDs was reflected in the change in the threshold voltage of the *I-V* characteristics induced on applying a magnetic field. The observed significant changes in the threshold voltage are attributable to the relative directions of magnetization between the upper and lower FePt-NDs, and a significantly high ON/OFF current ratio was also observed in the doubly-stacked FePt-NDs when switching the magnetization. This result is also related to the charging effect of the FePt-NDs.

The results obtained in this work show that the FePt-NDs are electrically charged and discharged as the direction of magnetization of the FePt-NDs changes, and the threshold voltage at which a current will flow is modified significantly by the direction of magnetization. This means that the FePt-NDs can be regarded as the gate of a spin diode with a sufficiently high ON/OFF current ratio, and the ON and OFF states can be switched by magnetization of the FePt, which is one of the most technologically important materials, since it has an extremely large magnetic anisotropy resulting in long term data retention even in an NDs structure. Furthermore, our samples are very compatible with Si-ULSI processing technology. We believe that our findings will open the way to constructing a spin diode/transistor that exhibits a high ON/OFF ratio, a large thermal stability sufficient for data retention, low power consumption, and room temperature operation.

In summary, we fabricated FePt-NDs with an areal density as high as ~10^11^ cm^−2^ on an ultrathin SiO_2_ layer and evaluated the local *I*-*V* characteristics through individual dots by using a magnetic cantilever under an external magnetic field. We confirmed at room temperatures that there is a clear change in the threshold voltage at which a current will flow through the FePt-NDs that depends on the relative directions of magnetization between the NDs and the magnetic cantilever. The origin of this significant change in the threshold voltage is considered to be the electrical charging of the FePt-NDs which varies with the direction of magnetization of the FePt-NDs. We also designed and fabricated doubly-stacked FePt-NDs with different coercivities between the lower and upper NDs, and confirmed a clear change in the threshold voltage at which a current will flow through the NDs that depends on the relative direction of magnetizations between the upper and lower dots. From the local *I*-*V* characteristics, which depend on the magnetization direction of the NDs, the FePt-NDs fabricated by a remote H_2_ plasma can be regarded as the gate of a spin diode with a significantly high ON/OFF current ratio. From a technological point of view, it is quite important that such a high ON/OFF current ratio could be achieved using FePt-NDs, which have a large magnetic anisotropy at room temperature, i.e., sufficient thermal stability for data retention, and are compatible with Si-ULSI processing.

## Methods

### Formation of FePt-nanodots

After the conventional wet-chemical cleaning steps for p-type Si(100) wafers, a ~1.7-nm-thick SiO_2_ layer was grown at 1000 °C. An Fe layer was first deposited uniformly on the SiO_2_ layer by electron beam evaporation and then covered uniformly with a Pt layer without exposure to air. Subsequently, the as-prepared ~1.7 nm-Pt/~1.4 nm-Fe bi-layer stack was exposed simply to a remote H_2_ plasma with no external heating^37^. The plasma was generated by inductive coupling with an external single-turn antenna connected to a 60-MHz generator through a matching circuit. The substrate was placed on the susceptor at a distance of 19 cm from the antenna to minimize ion damage. During the remote H_2_ plasma exposure, the gas pressure and VHF power were maintained at 13.3 Pa and 500 W, respectively.

### Formation of doubly-stacked FePt-nanodots

Doubly-stacked FePt-NDs were formed with lower and upper dots of different sizes. The dot size was controlled by changing the thickness of the Fe and Pt bi-layer stacks, e.g., FePt-NDs with an areal density of ~2.5 × 10^11^ cm^2^ and an average size of ~8.0 nm were formed by exposing a ~2.8 nm-Pt/~2.3 nm-Fe bi-layer stack to remote H_2_, which exhibited 2.5 kOe perpendicular coercivity and were used as upper dots. The lower dots were fabricated as described in the “Formation of FePt-nanodots” paragraph in this section, and had an areal density of ~4.5 × 10^11^ cm^−2^, an average size of ~5.0 nm, and a perpendicular coercivity of 0.5 kOe.

## Additional Information

**How to cite this article**: Makihara, K. *et al*. Nano spin-diodes using FePt-NDs with huge on/off current ratio at room temperature. *Sci. Rep.* **6**, 33409; doi: 10.1038/srep33409 (2016).

## Supplementary Material

Supplementary Information
