# Supplementary material for: Nano spin-diodes using FePt-NDs with huge on/off current ratio at room temperature
Source: Sci Rep. 2016 Sep 12;6:33409. doi: 10.1038/srep33409 (PMC5018842; doi:10.1038/srep33409)
Supplement: Supplementary Information [file srep33409-s1.doc]

**Nano spin-diodes using FePt-NDs with huge on/off current ratio at room temperature**

Katsunori Makihara^1*^, Takeshi Kato^1^, Yuuki Kabeya^1^, Yusuke Mitsuyuki^1^, Akio Ohta^1^, Daiki Oshima^1^, Satoshi Iwata^1^, Yudi Darma^2, 3^, Mitsuhisa Ikeda^1^ and Seiichi Miyazaki^1^

^1^Graduate School of Engineering, Nagoya University

^2^Department of Physics, Institut Teknologi Bandung, Bandung, Indonesia

^3^Singapore Synchrotron Light Source, National University of Singapore, Singapore

^*^E-mail address: makihara@nuee.nagoya-u.ac.jp

**Supplementary Information**

**[Fabrication of the FePt Nanodots]**

The FePt nanodots (NDs) were fabricated on p-type Si (100) wafers with a ~1.7-nm-thick SiO_2_ layer grown at 1000˚C in 2% O_2_ ambient. Before the formation of the FePt NDs, an Fe (~1.1 nm) layer was first deposited uniformly on the SiO_2_ layer by electron beam evaporation and then covered uniformly with a Pt (~1.4 nm) layer. Subsequently, the as-prepared Pt (1.4 nm) / Fe (1.1 nm) bi-layer stack was exposed simply to a remote H_2_ plasma (H_2_-RP) without external heating^36^. The plasma was generated by inductive coupling with an external single-turn antenna connected to a 60-MHz generator through a matching circuit. The substrate was placed on the susceptor at a distance of 19 cm from the antenna to minimize ion damage. During the remote H_2_ plasma exposure, gas pressure and VHF power were maintained at 13.3 Pa and 500 W, respectively. Before the H_2_-PR exposure, the Pt / Fe bilayer has a flat surface with a root mean square roughness of ~0.24 nm, which is almost identical to that for the as-grown SiO_2_ surface. On the other hand, after the H_2_-PR exposure for 10 min, the surface roughness significantly increased, indicating the formation of NDs structure as shown in Fig. 1. The areal density of the NDs was estimated to be ~4.8 × 10^11^ cm^-2^. Figure S1 shows the histogram of the dot heights measured by an atomic force microscope (AFM). We evaluated size distribution of the obtained FePt-NDs from the AFM topographic image as indicated in Fig. 1. The size distribution can be fitted to a log-normal function [s1] as shown in Fig. S1. From the curve fitting, average dot height can be roughly estimated to be ~8.7 nm. As shown in Fig. 1 (c), the in-plane and out-of-plane hysteresis loops of FePt-NDs showed similar shape to each other, indicating the perpendicular magnetic anisotropy of the FePt-NDs compensates the shape anisotropy. In order to investigate the origin of the perpendicular anisotropy, crystal structure of the FePt-NDs was characterized by X-ray diffraction (XRD) with Cu-*K*α radiation. Figure S2 shows XRD profiles of the as-deposited Pt/Fe bilayer and H_2_-RP exposed FePt-NDs. For the Pt/Fe bilayer, no obvious peaks except for the peak from the substrate were seen. On the other hand, the FePt 001 superlattice and 002 fundamental peaks were clearly seen for the sample after the H_2_-RP exposure, indicating the formation of (001) oriented L1_0_-FePt alloy. The L1_0_-FePt shows strong magneto-crystalline anisotropy with the easy axis along the *c*-axis. Thus the (001) orientation of the L1_0_-FePt is the origin of the perpendicular anisotropy observed in FePt-NDs shown in Fig. 1.

**Figure S1 | Size distribution of FePt-NDs.** The dot size distribution evaluated from AMF image taken after H_2_-RP. The corresponding curve denotes the log-normal functions well-fitted to the measured size distribution.

[s1] Irani, R. R. & Callis, C. F. Particle size: measurement, interpretation, and application. Wiley, New York (1963).

**Figure S2 | XRD patterns of Pt/Fe bi-layer and FePt-NDs.** The XRD pattern of FePt-NDs showed distinct diffraction peaks due to FePt (001) and (002) planes, indicating the formation of (001) oriented L1_0_-FePt phase.

**[Local I-V characteristics of the FePt and Pt Nanodots]**

Local *I*-*V* characteristics of FePt-NDs prepared by the RP-H_2_ exposure was measured by using an AFM cantilever (tip) under external magnetic fields. The cantilever was contacted to the sample surface and was kept at a certain position to inject electrons from the probe to FePt-NDs during the *I*-*V* measurement. As discussed in Fig. 2, when a magnetic CoCrPt-coated AFM tip was used for the *I*-*V* measurements, a significant variation of the threshold voltage to flow the current of 1 nA was observed by changing the relative direction of magnetizations of FePt-NDs and CoCrPt tip. When the magnetizations of FePt and CoCrPt became antiparallel, the threshold voltage was –4.3 V, while the voltage reduced to –1.2 V when they were parallel. The ON/OFF current ratio at 1.5 V was estimated to be 10^2^, which is extremely large compared to the reported spintronics devices, such as spin FET, spin valve transistor, and etc. More importantly, we confirmed such a large ON/OFF current ratio by using FePt, which is one of the most technologically important materials. In order to check that our findings are originated from the spin dependent transport effect via FePt-NDs, we carried out several experiments to rule out other possibilities.

Figure S3 shows local *I*-*V* characteristics of FePt-NDs measured by using a non-magnetic Rh-coated AFM tip under external magnetic fields. The magnetic field was applied by placing NdFeB magnet under the sample (see inset in Fig. S4). Here we defined the threshold voltage at a certain current determined by considering the current level of each sample in the measured voltage range, which depends on the work function of AFM tips and the thickness of underlying SiO_2_. Therefore, it is difficult to directly compare threshold voltages between I-V characteristics for measurements with different AFM tips or different samples. As shown in the figure, no obvious change in the *I*-*V* characteristics was seen under the external field of ±4.5 kOe. Similarly, no obvious change of the *I*-*V* characteristics was seen for Pt-NDs flowing the current using magnetic CoCrPt-coated tip. Figure S4 shows the results obtained for Pt-NDs. In the case of the Pt-NDs, a ~3.6-nm-thick SiO_2_ layer was thermally grown on a p-Si(100) substrate. After that, a ~1.2-nm-thick Pt film was evaporated on the SiO_2_ by electron beam without any extra heating. Subsequently, the Pt films were exposed to a remote plasma of pure H_2_ under the same experimental condition with the case of FePt-NDs formation. The CoCrPt-coated tip was initially magnetized upward direction, and contacted on Pt-NDs to inject electrons from tip to NDs. Before the application of the field, the threshold voltage to flow the current of 0.1 nA was –4.8 V. After the application of the external field of 4.5 kOe to switch the magnetization of CoCrPt, no obvious change of the threshold current was confirmed. This experiment also shows that the magneto-static interaction between magnetic tip and magnet placed under the sample does not change the local *I*-*V* characteristics discussed in this paper.

**Figure S3 | Local I-V characteristics of FePt-NDs measured with a Rh-coated tip.** When the non-magnetic metal coated AFM tip was contact to the sample and I-V curves were measured under magnetic fields with two opposite directions, no significant change in the I-V curve from the case without magnetic field was detected.

**Figure S4 | Local I-V characteristics of Pt-NDs.** We also fabricated Pt-NDs from the ultrathin Pt films exposed to H_2_-RP, as reported in ref.38, and measured I-V curves by a CoPtCr-coated tip with and without the magnetic field application. No significant difference in the I-V curves was also obtained under the same experimental setup. An AFM image of Pt-NDs is also shown in the inset.

**[Force curve characteristics of the FePt]**

To examine an effect of magnetic force on the local electron transport properties, we evaluated an impact of the external magnetic field on the interaction between the tip and sample surface. Figure S5 shows the typical force curves of CoCrPt-coated tip taken with and without magnetic field at 4.5 kOe. The solid circles and lines represent the force curves with and without external field, respectively. The force curve measurement starts with the tip position of 200nm. As the sample approaches to the cantilever tip, the cantilever bends toward the sample due to adsorbed water molecules or attractive surface forces and then contacts at a position of ~50 nm. With further movement of the sample toward the tip, the cantilever is pushed back over its original rest position and bends upward. With moving the sample back, the bending of cantilever reduces and then changes to downward until the cantilever separates suddenly and returns to its rest position. The same experiment was carried out applying a magnetic field by placing a magnet under the sample, and no obvious change of the force curve was confirmed; the tip touches to the sample at a position of ~ 50 nm irrespective of the external field. This result support the magneto-static interaction between magnet and magnetic tip does not change local I-V characteristics as discussed in Fig. S4. Notice that, for the case of electric field application, we can clearly see significant change in the force curves as shown in Fig. S5 (b). In this experiment, we measured the force curves of Si quantum dots covered with Au film formed on SiO_2_/Si substrate where Al back contact was formed as schematically illustrated in the inset of Fig. S5 (b), under the negative bias application to the Al bottom electrode with respect to the grounded Au top electrode. When applying a bias of -15V to the substrate, the AFM cantilever started to bend toward the sample at a tip position of ~150 nm which is more distant in comparison with that without bias application. This result indicates the significant effect of electrostatic force on the force between the tip and sample surface. From these results, we can conclude that both magnetic NDs and tips are necessary to observe the significant change of the threshold voltage by applying a magnetic field and the ON/OFF state was controlled by the relative direction of the magnetizations of magnetic NDs and magnetic tip.

**Figure S5 | Force curve under the magnetic field and electric field application.** a. Force curves of FePt-NDs measured by using a magnetic metal coated AFM tip with and without magnetic field application at 4.5 kOe. b. Force curves of Si quantum dots covered with Au films measured by using a metal coated AFM tip under the negative bias application at -15 V to the bottom electrode of Si-substrate with respect to the grounded Au top electrode.
